# Therapeutic challenges in peripheral T-cell lymphoma

**DOI:** 10.1186/s12943-023-01904-w

**Published:** 2024-01-04

**Authors:** Yunpeng Luan, Xiang Li, Yunqi Luan, Junyu Luo, Qinzuo Dong, Shili Ye, Yuejin Li, Yanmei Li, Lu Jia, Jun Yang, Dong-Hua Yang

**Affiliations:** 1 The First Affiliated Hospital of Yunnan University of Traditional Chinese Medicine, Kunming, 650021 China; 2 Key Laboratory for Forest Resources Conservation and Utilization in the Southwest Mountains of China, Ministry of Education, Southwest Forestry University, Kunming, 650224 China; 3 NMPA Key Laboratory for Safety Research and Evaluation of Innovative Drugs, Beijing Key Laboratory of Analysis and Evaluation On Chinese Medicine, Beijing Institute for Drug Control, Beijing, 102206 China; 4 The Affiliated Hospital of Kunming University of Science and Technology, Kunming, 650032 China; 5 New York College of Traditional Chinese Medicine, 200 Old Country Rd, Suite 500, Mineola, NY 11501 USA

**Keywords:** Leukemia, Lymphoma, PTCL, NHL, T-ALL, ALCL, TME, Relapse, Refractory, Drug resistance, Clinical trial

## Abstract

Peripheral T-cell lymphoma (PTCL) is a rare and heterogeneous group of hematological malignancies. Compared to our knowledge of B-cell tumors, our understanding of T-cell leukemia and lymphoma remains less advanced, and a significant number of patients are diagnosed with advanced stages of the disease. Unfortunately, the development of drug resistance in tumors leads to relapsed or refractory peripheral T-Cell Lymphomas (r/r PTCL), resulting in highly unsatisfactory treatment outcomes for these patients. This review provides an overview of potential mechanisms contributing to PTCL treatment resistance, encompassing aspects such as tumor heterogeneity, tumor microenvironment, and abnormal signaling pathways in PTCL development. The existing drugs aimed at overcoming PTCL resistance and their potential resistance mechanisms are also discussed. Furthermore, a summary of ongoing clinical trials related to PTCL is presented, with the aim of aiding clinicians in making informed treatment decisions.

## Introduction

**Fig. 1:**
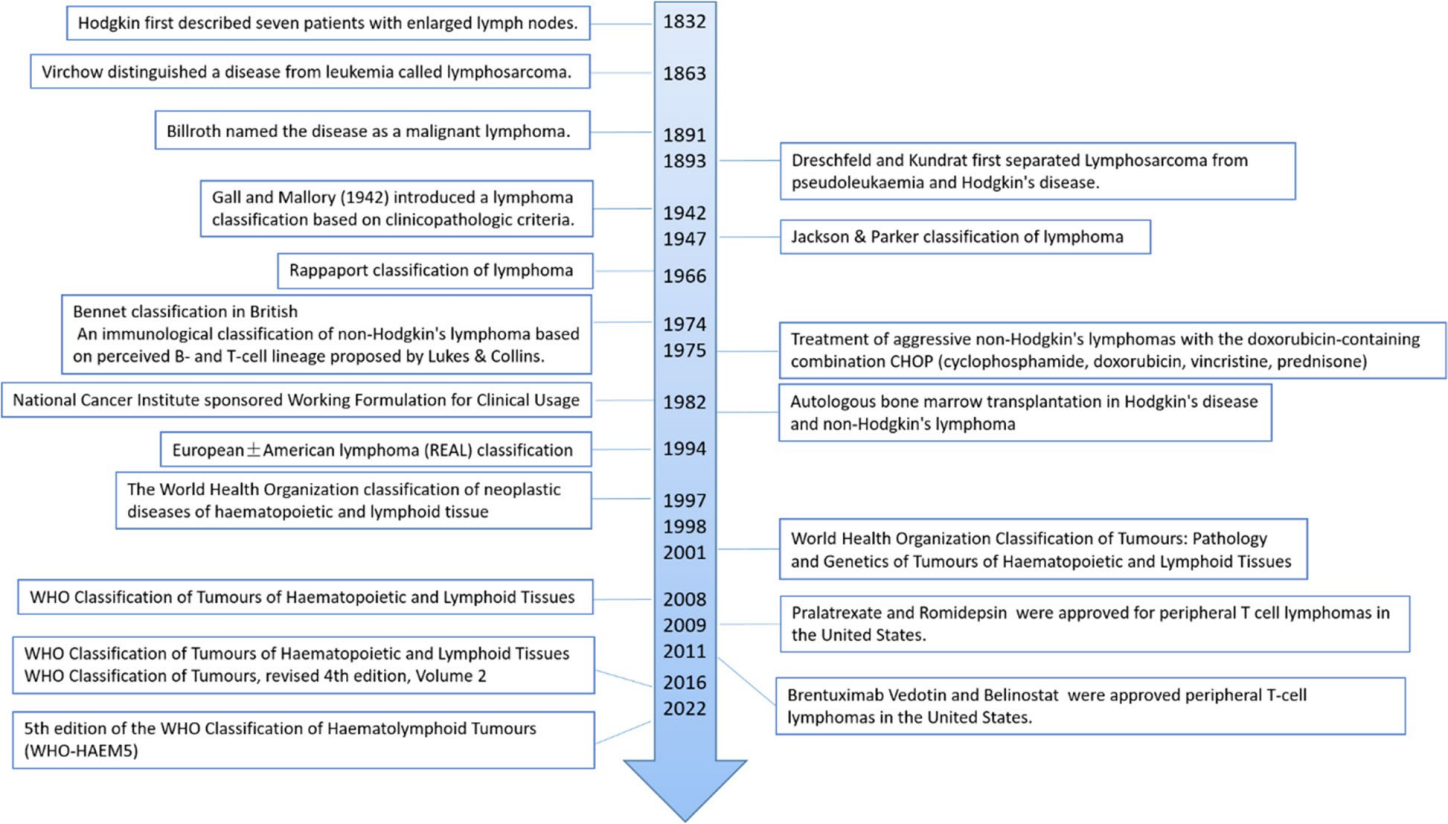
The history of our understanding and therapeutic choice of PTCL [4, 5]

Peripheral T-cell lymphoma (PTCL) is a rare but heterogeneous group of hematological malignancies. This group of mature T-cell non-Hodgkin's lymphomas (NHL) is an aggressive disease associated with poor prognosis. PTCL accounts for 5–10% of all non-Hodgkin's lymphomas [1]. Our understandings on T-cell leukemia and lymphoma lag behind that of B-cell tumors, and a large proportion of patients have advanced disease at diagnosis. The history of our understanding and choices of PTCL is summarized in Fig. 1. The World Health Organization (WHO) categorizes PTCL into approximately 30 different types. Broadly speaking, nodal, extranodal, and leukemic PTCL typically manifest as aggressive diseases, with a five-year survival rate of about 30%. On the other hand, cutaneous PTCL tends to present as a more slow-growing disease [2]. The WHO classification of haematolymphoid tumors (WHO-HAEM5) presents new molecular and histopathological findings that facilitate the diagnostic classification of this type of tumor [3].

The incorporation of CD20-based combination chemotherapy has improved the long-term survival rate of diffuse large B-cell lymphoma, now reaching an impressive range of 60- 70%. However, regimens that have been successfully used in many aggressive B-cell lymphomas (i.e., anthracycline-based multiagent regimens, including CHOP) are not as effective in T-cell lymphomas [6, 7]. Approximately 70% of patients with PTCL develop relapsed or refractory disease after the first-line therapy. Attempts to improve outcomes by "adding" or "substituting" anthracyclines have consistently failed, and most patients with T-cell lymphoma will die from lymphoma- or treatment-related complications within a few years after diagnosis [8]. Undoubtedly, addressing most T-cell lymphomas has proven to be a difficult and unmet medical challenge. This article thoroughly examines the significant impact of tumor heterogeneity and the tumor microenvironment (TME) on treatment resistance in PTCL. Additionally, it presents an up-to-date analysis of the molecular mechanisms contributing to treatment resistance in PTCL. Finally, potential strategies to both prevent and overcome treatment resistance in PTCL are discussed.

### Sources of treatment resistance in PTCL

The occurrence of relapsed or refractory disease in PTCL is highly prevalent due to the emergence of drug resistance during treatment. Despite the availability of various treatment options, none have been universally curative, and eventually, drug resistance may develop in response to available treatments [9]. Although these two phenomena can coexist, intrinsic treatment resistance is often distinguished from acquired resistance. Specifically, intrinsic resistance arises from the disease itself and easily leads to treatment refractoriness, whereas acquired resistance arises from the acquisition of resistance-mediated features through mutation or non-mutation during treatment and often leads to disease relapse [10, 11]. In PTCL, treatment resistance often arises from a cancer ecosystem composed of multiple sources, including the heterogeneous cancer cells themselves and their surrounding tumor microenvironment (TME).

#### Heterogeneity of tumors

**Fig. 2:**
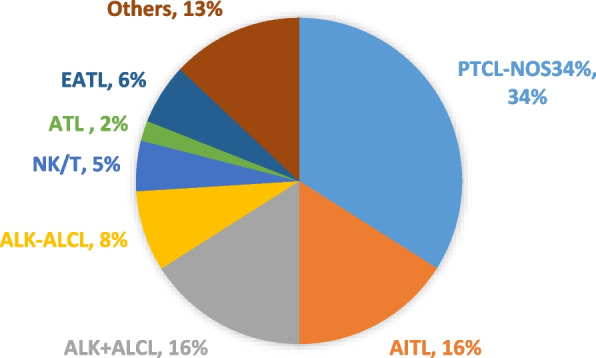
Incidence of different Peripheral T-cell lymphoma subtypes according to IPTCLP [7]

PTCL represents a remarkably heterogeneous group of diseases, characterized by the absence of distinct molecular markers and morphological features [12]. T-cell lymphomas consist of a variety of rare diseases that can be classified as indolent or aggressive and account for 12% of all NHL. In 2016, the World Health Organization (WHO) released a revised classification of T cell/NK cell lymphoma, categorizing it into two main groups: precursor T cell tumors and mature T cell tumors. The mature subgroup was further sub-divided into leukemic, intranodal, extranodal, and cutaneous types. Additionally, lymphoma can be further classified into indolent (slow-growing) disease and aggressive forms [2]. Indolent lymphomas are characterized by a long disease course and are usually resistant to standard chemotherapy, whereas aggressive lymphomas usually have an acute presentation with B symptoms(such as weight loss, night sweats, and fever) and rapid progression. Cutaneous T-cell lymphoma (CTCL) is considered indolent, whereas peripheral mature lymphoma (PTCL), including other types, is considered aggressive. PTCL-nos (not otherwise specified) is the most common PTCL, followed by anaplastic large cell lymphoma (ALCL) and angioimmunoblastic T-cell lymphoma (AITL) [13, 14] (Fig. 2). The 2022 update of the WHO classification of lymphoid neoplasms of blood (WHO-HAEM5) includes new insights into pathogenesis and molecular genetics as well as new concepts underlying the classification [3].

T lymphoblastic leukemia/lymphoma (T-ALL/LBL) can present as lymphoma, often accompanied by rapid enlargement of mediastinal lymph nodes and pleural effusion, and can also be accompanied by leukemia of blood and/or bone marrow [15, 16]. In T-ALL /LBL, the most common genetic alteration in 50 to 70% of patients is NOTCH1 activating mutations. NOTCH1 signaling is required to induce maturation of immature lymphoid precursor cells into T cells, and through NOTCH1 activation, MYC is activated [17, 18]. In addition to NOTCH1 activation, TAL1 activation is another common driver of tumorigenesis, occurring in 25% to 35% of T-ALL/LBL cases [19]. CDKN2A/B gene deletion occurs in up to 70% of T-ALL/LBL patients [17]. NUP214 fusion, PTEN and WT1 deletion or mutation, PHF6 deletion and other genetic changes can occur in some T-ALL/LBL patients [20–22]. In contrast, early T-cell precursor lymphoblastic leukemia (ETP-ALL) harbors mutations commonly found in myeloid tumors, including alterations in FLT3, DNMT3A, NRAS, KRAS, IDH1, or IDH2, all of which are genetically rare in classical T-ALL/LBL [23].

Although all or even most cases of systemic EBV + T-cell lymphoma (sEBV + TNHL) do not have specific chromosomal abnormalities, there are some common features: cases with alterations usually have complex karyotypes (3 or more chromosomal alterations), and nearly 40% of cases in the literature have add(9)(p24) [24]. In addition, variants in chromosomes 1, 7, 11, 17, 20, 21, and X are present in more than 20% of reported cases, whereas DDX3X, BCOR/BCORL2, and TET2 are present in 20% of chronic active EBV disease [25, 26]. Chromosomal alterations associated with CAEBV in HV-LPD patients include 6q deletion or 6p gain. These molecular and chromosomal abnormalities are also seen in extranodal nasal-type NK/ T-cell lymphoma. A study of primary EBV-positive T/ NK-cell lymphoma showed that approximately 20% of the cases had recurrent copy-number aberrations, including deletions of chr14q11.2(100%), chr3q26.1(67%), and chr22q11.23(33%) [27, 28].

Extra-nodal NK/T-cell lymphoma (ENKTL) can be divided into three different subtypes with different molecular characteristics and treatment outcomes (3-year OS rates were 79.1%, 91.7% and 38.5%, respectively) [29]: TSIM (tumor-suppressor/immune-modulator) subtype is associated with JAK/ STAT pathway activation, NK-cell origin, TP53 mutations, genomic instability (including 6q21 deletion and 9p24.1 and/or 17q21.2 amplifications), and PD-L1/2 overexpression [30]; HEA (HDAC9-EP300-ARID1A) subtype is associated with epigenetic changes through HDAC9, EP300 and ARID1A mutations, NF-KB activation, T-cell origin, and T-cell receptor signaling activation; Finally, MB (MGA-BRDT)subtype is associated with MYC overexpression and poor prognosis [29, 31].

ATXN1 or CIC alterations are present in 53% of adult T-cell leukemia/lymphoma (ATLL) cases, and CCR4 (C–C chemokine receptor 4) mutations are common, and the majority of ATLL patients exhibit CCR4 overexpression, which is associated with skin involvement and worse prognosis [32]. 9p24 amplification with PDL1 amplification occurs in 10 to 20% of cases, and TP53 mutations occur in 16% of cases, both of which are associated with a poor prognosis [33].

The most common translocation in anaplastic large cell lymphoma(ALCL) is t(2; 5) (p23; q35), NPM1::ALK is found in approximately 85% of ALK + ALCL [12]. The prognosis of ALK + ALCL is better than that of ALK-ALCL, and the 5-year overall survival rate is 70–90% [34]. Alk-negative ALCL cases have considerable genetic heterogeneity, and DUSP22 gene rearrangement is found in approximately 30% of systemic ALK-negative ALCL patients [35]. Certain studies have proposed that dusp22 rearrangement in ALK-negative ALCL demonstrates a favorable outcome, similar to that of ALK-positive disease. However, other studies have indicated a more aggressive disease course compared to ALK-positive disease [35, 36]. TP63 rearrangement occurs in about 8% of Alk-negative ALCL cases and is associated with poor prognosis, with a 5-year overall survival rate of only 17%, which is lower than that of PTCL-NOS [37]. PTCL-NOS is a T-cell lymphoma that does not meet any of the specific subtype criteria. This tumor is also the most common type of T-cell lymphoma, accounting for 25–30% of all cases [38]. The prognosis of PTCL-NOS relies on its genetic heterogeneity, leading to its classification based on gene expression profiles: one with high expression of GATA3 (PTCLGATA3) and the other with high expression of TBX21 (PTCL-tbx1) [39]. PTCL-GATA3 is linked to PI3K/mTOR pathway activation, exhibiting a more aberrant genome, and thus, associated with a poor prognosis, with a 5-year overall survival rate of 19%. In contrast, PTCL-TBX21 is characterized by NFKB pathway activation, fewer genomic abnormalities, and a more favorable prognosis, with a 5-year overall survival rate of 38% [40]. PTCL-NOS cases with TP53/CDKN2A alterations show considerable chromosomal instability and a poor overall survival rate, exhibiting an inverse correlation with TFH marker expression. The majority of PTCL-NOS cases have a homozygous deletion of CDKN2A, which appears to be linked to an unfavorable prognosis [25].

In the latest WHO classification, Tfh (T follicular helper) PTCL encompasses AITL with Tfh phenotype, follicular PTCL, and nodal PTCL, which are tumors with similar clinical presentation, gene expression, DNA copy number, abnormalities, and mutational profiles [2]. 3, 5, 21, and X chromosome gain/trisomy are the most common cytogenetic abnormalities in AITL. Other visible chromosomal alterations include increases in 11q13, 19, or 22q and loss of 13q10. Mutations in genes observed in AITL overlap for the most part with those observed in medullary tumors [22].TET2 mutations are seen in 47% ~ 83% of AITL cases, and DNMT3A alterations are seen in 26% ~ 38% of cases. These genes are involved in epigenetic regulation, and their mutations lead to 5-hydroxymethylcytosine loss through a common mechanism in PTCL. There are also IDH2 mutations seen in 20–45% of AITL, and RHOA p.G17V mutations are seen in 70% of AITL [41–43]. The fact that RHOA and IDH2 mutations are confined to tumor cells suggests that they may be the second strike in a multistep oncogenic process [44]. These mutations are also present in Tfh-type PTCL, with the exception of the IDH2R172 mutation, which is strongly associated with AITL labeling and correlates with specific pathological manifestations, and the SYK fusion, which is more frequently detected in follicular PTCL [45, 46].

#### Tumor microenvironment

Lymphoma occurrence is not solely driven by tumor-autonomous processes; rather, it requires the intricate interaction of the tumor microenvironment (TME). The TME encompasses tumor cells, immune cells, stromal cells, blood vessels, and the extracellular matrix surrounding the tumor. Interactions between stroma cells and tumor cells within the TME, along with the secretion of soluble factors, have been identified as contributors to treatment resistance in various cancer types [47, 48]. As PTCL is relatively rare, our understanding of the PTCL TME is still in its early stages, with most knowledge derived from B-cell NHL experiences.

Programmed cell death ligand I (PD-L1) expression was more pronounced in the TME of PTCL, seen in 73% of CTCL cases and 39% of other PTCL cases. PD-L1 is also highly expressed in malignant cells such as nasal NK/ T cell lymphoma (NKTCL) and extranasal NKTCL. Almost all EBV-associated lymphomas are associated with high PD-L1 expression [27]. Serum PD-L1 level is associated with the prognosis of ENKTCL. For example, Nagato and colleagues reported that elevated levels of PD-L1 in tumor cells is correlated with elevated levels of PD-L1 in serum and worse OS, which is associated with immune escape [47]. As previously described, GATA3 expression in PTCL-NOS is associated with a poor prognosis [39] and is characterized by type 2 helper t cell (Th2) -related cytokines, including interleukin (IL)-4, IL-5, IL-10, and IL-13 [48], which promote macrophage polarization to alternative M2-type macrophages [49]. M2 macrophages promote angiogenesis by secreting pro-angiogenic cytokines such as VEGF. In addition, they secrete IL-10 and transforming growth factors, which up-regulate the expression of PD-L1 on macrophages in an autocrine manner. Binding of PD-L1 to PD-1 expressed on T cells results in suppression of T cell function and, consequently, immunosuppression [50, 51]. Clinic pathological correlation studies have provided evidence for the prognostic significance of tumor-associated macrophages in T-cell lymphoma [52]. Among 64 T-cell lymphomas, high tumor-associated macrophage content was associated with poor prognosis in multivariate analysis (high macrophage content vs low macrophage content, overall survival, OS: 28.1% vs 44.3%) [53]. The degree of macrophage infiltration is also inversely correlated with survival, with multiple studies showing that a high content of CD163-positive macrophages is associated with low survival [54, 55].

The infiltration of other non-neoplastic T cells in T-cell lymphomas may also regulate TME. Regulatory T cells (Tregs) are a subset of CD4-positive T cells that inhibit immune responses and maintain immune tolerance. Tregs are characterized by high levels of CD25 and forkhead transcription factor FOXP3 [56, 57]. Tregs in the lymphoma microenvironment may suppress immune-mediated antitumor responses, thereby enhancing tumor cell survival. However, Tregs may also down-regulate the inflammatory response in the microenvironment and promote tumor progression, thereby inhibiting tumor cell proliferation. The opposite regulatory effects of Tregs on tumor cells may explain the apparently contradictory prognostic effects of Tregs on different types of T-cell lymphomas. The number of Tregs remained an independent prognostic biomarker in multivariate analysis, and a large number of Tregs infiltration was associated with higher survival [58, 59].

#### Multiple drug resistance

Drug resistance is a common and difficult obstacle in the treatment of mature T/ NK-cell lymphoma, and multidrug resistance (MDR) phenotype is considered to be one of its mechanisms [60]. MDR refers to the acquired cross-resistance to a variety of structurally and functionally unrelated drugs. MDR is often associated with increased expression of drug efflux transporters of the ATP-binding cassette (ABC) protein family [61, 62]. The ABC transporter superfamily contains 48 ABC transporters, which are divided into seven subfamilies based on sequence homology and protein organization. Accumulating evidence suggests that ABC transporters play a key role in the physiological transport and export of drugs and toxic substances, which can export a variety of chemotherapeutic agents outside of cells [63]. T/ NK-cell lymphoma cases showed a high frequency of MDR protein expression [64]. In earlier reports, poor response to chemotherapy in ATL patients was partly due to high expression of P-gp or MDR1. In one study, the expression of P-glycoprotein (P-gp), multidrug resistance-related protein 1 (MRP-1), breast cancer resistance protein (BCRP), and lung resistance protein (LRP) in 45 cases of mature NK/ T-cell lymphoma was examined by immunohistochemistry. The positive rates of P-gp, MRP-1, BCRP and LRP were 31% (13/42), 74% (31/42), 78% (32/41) and 59%(26/44), respectively [65]. Jung et al., in a study of drug resistance in T—and NK-cell lymphomas, reported a statistically significant association with treatment failure and overall survival [66]. Yamaguchi et al. also reported high P-gp expression in their study of nasal NK-cell lymphoma patients and suggested that the poor prognosis of these patients may be related to P-gp expression [67]. Egashira et al. reported that P-gp expression in CD56-positive NK-cell tumors was associated with poor prognosis [68].

It has been confirmed that in B-cell lymphoma, the five drugs containing R-CHOP do not show synergistic effect, but cross-resistance is very low, indicating that the efficacy of RCHOP is produced by the combination of non-overlapping active drugs, and the reason for the poor efficacy of CHOP against ENKTL is the expression of the multidrug efflux pump P-glycoprotein /MDR1 [69].The high expression of P-glycoprotein in PTCL lymphoma cells, doxorubicin, vincristine, and prednisone, which are substrates of Pgp, easily induces intrinsic resistance to drugs due to the upregulation of P-gp expression [70]. ABCC4 and ABCG4 were significantly up-regulated in human NK/T cell lymphoma YTS and SNK-6 cells compared with normal NK cells [71]. Overall, it is important to highlight the role of ABCC4 in drug resistance. Based on gene expression regulation technology, overexpression of ABCC4 and ABCG4 can induce epirubicin (EPI) and cisplatin (DDP) resistance in human NK/ T cell lymphoma YTS cells and reduce cell apoptosis [72]. Meanwhile,IL-6, IL-10, and IL13 mediate ABCC4 resistance in T-cell lymphoma [64, 73, 74].

#### Signaling pathways in PTCL

**Fig. 3:**
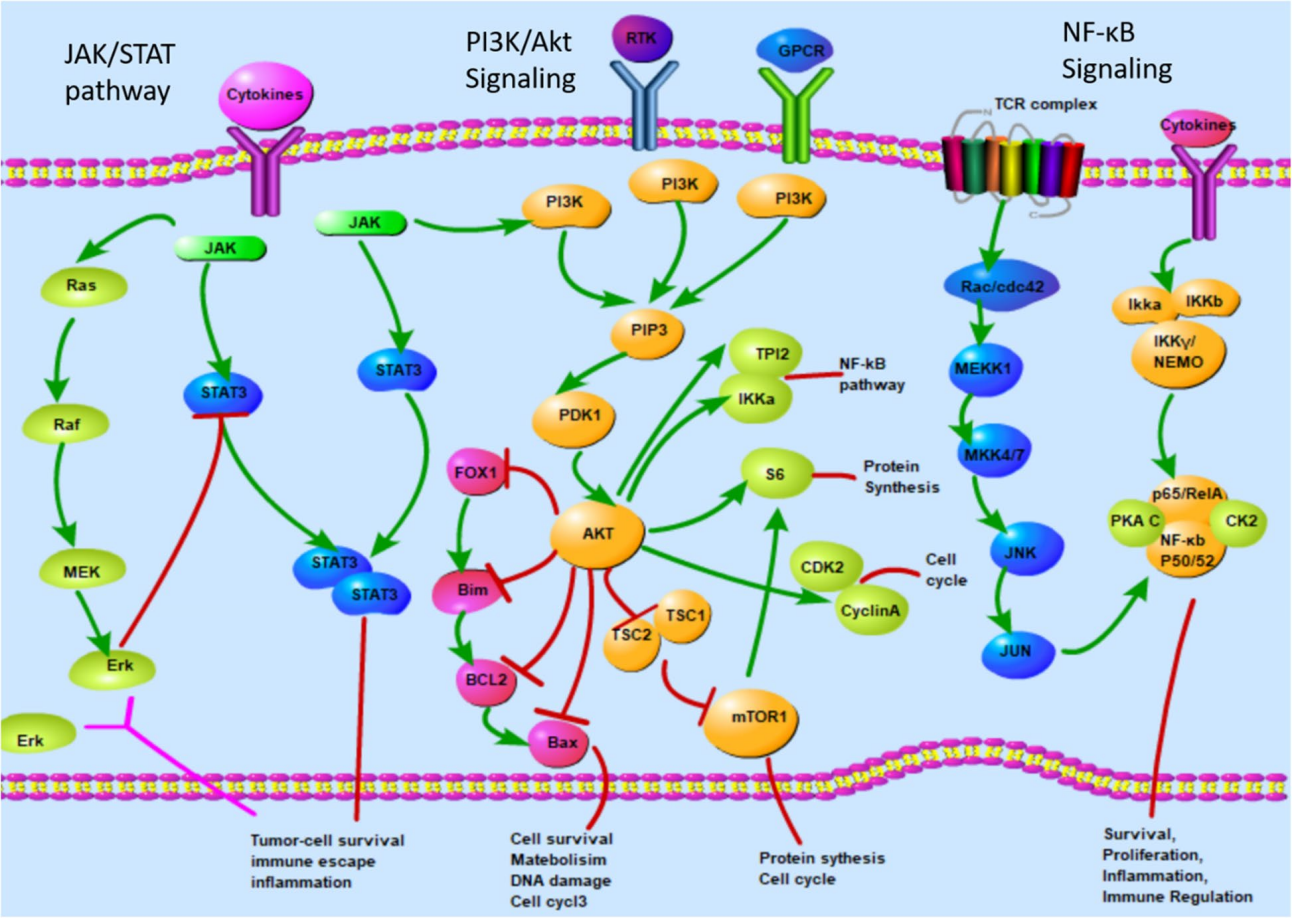
Part of the pathways, such as NF-κB, JAK/ STAT3 and PI3K/AKT signaling pathways in the pathogenesis of PTCL

The mechanism of drug resistance of lymphoma is closely related to the signaling pathways of lymphoma cells. Recent genetic analyses of PTCL have improved our understanding of the pathogenesis of this malignancy. The activation mutation of NF-κB, Notch, JAK/ STAT3, RHOA and PI3K/AKT signaling pathways play an important role in the pathogenesis of PTCL [29, 75]. The expression of JAK/STAT pathway genes is upregulated in ENKTL, and mutations in JAK3, STAT3, and STAT5B lead to constitutive activation of the JAK/STAT pathway, which occurs on the transcription factor jak3 in about 35% of cases, resulting in severe immunodeficiency characterized by a lack of T and NK cells [76, 77]. DDX3X, the RNA helicase gene, which mutated in 20% of PTCL, resulting in cell cycle arrest and loss of transcriptional activation of the nuclear factor κB (NF-κB) and mitogen-activated protein kinase (MAPK) pathways. Clinically, the presence of DDX3X mutation indicates a poor prognosis [78, 79]. PTPRK is known to dephosphorylate phosphorylated stat3, resulting in its inactivation. Loss of PTPRK and low expression of PTPRK due to aberrant promoter hypermethylation can cause constitutive activation of STAT3, leading to proliferation and development of PTCL. Downregulation of PTPRK is associated with advanced disease and poor outcomes in patients treated with the steroids, methotrexate, ifosfamide, L-asparaginase, and etoposide (SMILE) regimen [80]. NF-κB is involved in pro-proliferative signal transduction in a variety of lymphoid malignancies [75, 81]. Although the mechanism needs to be further investigated, GEP studies suggest increased expression of NF-κB-related genes in PTCL and that NF-κb inhibitors induce apoptosis in PTCL cells [82], findings that support the hypothesis that this pathway plays an important role in PTCL. In addition, NF-κB is involved in the pathogenesis of hemophagocytosis, which is the main cause of death in PTCL patients [83].(Fig. 3). In addition, increasing data suggest that viral components are involved in multidrug chemotherapy resistance in lymphoma cells, and several mechanisms may be associated with oncogenic viral-mediated chemotherapy resistance, which is caused by changes in disease signaling pathways [84]. Under latent EBV infection, intracellular ROS production increases P-gp expression via the STAT1 pathway, and ROS scavenger NecroX-5 down-regulates ROS, effectively attenuating P-gp-associated chemotherapy resistance in EBV-positive NK/ T-cell lymphoma. LMP1 and/or other viral components are also involved in P-gp-dependent chemoresistance [28, 85].

### Epigenetics in PTCL

Epigenetics is the stable clonal inheritance of expression states that cannot be explained by DNA variants. Gene expression is typically maintained through DNA methylation and post-translational methylation, acetylation, phosphorylation, and ubiquitination of histone and non-histone proteins [86]. Mutations in genes encoding proteins involved in epigenetic regulation are seen in various malignancies including PTCL, particularly in those expressing follicular helper T cell (TFH) differentiation markers, such as angioimmunoblastic T-cell lymphoma (AITL) and some PTCL-NOS [87]. Disruption of DNA methylation and histone modifications has become a hallmark of these diseases and the basis for epigenetically targeted therapies [88]. As the understanding of epigenetic complexity continues to deepen, it has been found that mutations in these epigenetic regulators have a global impact on lymphoma development and drug sensitivity, and the ability to silence multiple genes at the same time through the regulation of a large number of genes leads to polygenic drug resistance [89, 90].

#### MicroRNAs in PTCL

MicroRNA is a type of RNA with length of 19 to 22 nucleotides, which is involved in important biological processes such as development, proliferation, differentiation and apoptosis [91]. Recent studies have shown that the differentiation of various T cell subsets is regulated by multiple miRNAs targeting different signaling pathway proteins/molecules, resulting in initiation or inhibition/termination of differentiation [92, 93]. Different microRNAs are uniquely expressed in lymphoid T cells and play a role in the development and differentiation of various subtypes by targeting their target genes. Aberrant expression of miRNAs may be involved in T-cell leukemia and lymphopoiegenesis and may function as tumor suppressor genes such as miR-451, miR-31, miR-150, and miR-29a or oncogenes such as miR-222, miR-223, miR-17–92, and miR-155. In T-cell leukemia and lymphoma, microRNA can be used as novel biomarkers for prognosis and diagnosis, or as indicators of disease severity [94, 95].

The increased expression of miR-122 is associated with poor prognosis in advanced mycosis fungoides (MF). The up-regulation of miR-21, miR-486 and miR-214 is involved in promoting SzS cell survival and is involved in the apoptotic resistance of CTCL cell lines, and may even become incurable CTCL [96]. The expression levels of miR-21 and miR-155 in NK cell lymphoma cell lines were significantly higher than those in normal NK cells [97]. Plasma miR-221 level may have diagnostic and prognostic significance in extranodal NK/ T cell lymphoma (ENKTCL) [98]. In fact, there is an indirect relationship between miR-221 expression and overall survival after treatment of this lymphoma. Evaluation of miR-146a expression in NKTL tissues showed that patients with low levels of miR-146a expression were associated with chemotherapy resistance and poor prognosis. This miRNA acts as a tumor suppressor and therefore can be used for prognosis of PTCL. Evaluation of the expression level of miR-16 in paraffin-embedded lymph node samples of T lymphoblastic lymphoma/leukemia (T-LBL/ALL) patients showed that the overall survival of patients with high levels of miR-16 was higher than that of patients with low levels of miR-16, and it can be used as a prognostic marker for T LBL/ALL108 patients [99]. Elevated exosomal miR-4454, miR-21-5p, and miR-320e levels were associated with poor overall survival. Elevated levels were also found in patients who relapsed after treatment. These three miRNAs were overexpressed in PTCL cell lines resistant to etoposide [100].

### Approved and emerging therapies for R/R PTCL

The exploration of treatment for PTCL is very active, and the development of treatments has been driven primarily by advances in the understanding of the biology of the disease.

#### CD30 monoclonal antibody agents

CD30 receptors are expressed in Hodgkin's disease, T-cell lymphoma subsets, and activated T cells [101]. In 376 PTCL patient samples, CD30 was expressed in 58% of PTCL-NOS, 63% of AITL, and almost 100% of ALCL. Similar to the therapeutic effect of CD20 monoclonal antibodies in B-cell lymphomas, there is an increasing interest in targeting CD30 as a potential therapeutic option for PTCL [102]. Brentuximab Vedotin (BV) is an antibody–drug conjugate in which an anti-CD30 monoclonal antibody is coupled to the tubulin toxin monomethyl lauristatin E (MMAE) [103]. Once bound to the CD30 receptor, it is internalized and causes disruption of microtubule polymerization and cell death. BV-CHP has shown clinical benefit in ALCL, making it the preferred option for the treatment of this disease, and it is strongly considered for other CD30 + PTCL [8]. In a large prospective phase II study and another large phase III randomized study (ECHELON-2), BV combined with other chemotherapy agents showed good therapeutic effect, and BV also achieved good efficacy in relapsed and refractory PTCL as a single agent [104–106]. At ASH 2022, the updated 5-year ECHELON-2 clinical trial showed that compared to CHOP, first-line treatment of PTCL patients with A + CHP continued to provide clinically meaningful improvements in PFS and OS with a manageable safety profile, with 5-year PFS rates at a median follow-up of 47.6 months in the A + CHP group and CHOP group of 51.4% and 43.0%, with 5-year overall survival rates of 70.1% and 61.0% and a 28% reduction in the risk of death (HR = 0.72; 95% CI: 0.53–0.99), respectively. Among patients treated with vibutuximab after relapse, the objective remission rate was 59% for re-treatment with BV after A + CHP and 50% for re-treatment with vibutuximab after CHOP. In ECHELON-2, patients over 65 years of age who received a + CHP had significantly improved outcomes. An ongoing phase 2 trial is providing additional data on BV retreatment in patients with classical Hodgkin's lymphoma, sALCL, or other cd30-positive PTCL (NCT03947255) [107]. Although BV has high activity in R/R HL, most patients eventually develop BV resistance. It was reported that antigen downregulation is a potential resistance mechanism to any antigen-targeted therapy [108], however, it seemed CD30 loss does not appear to be a common event in BV-refractory HL. The investigators found that CD30 expression was maintained in tumor samples from patients with BV-refractory HL, as well as in two independent BV-resistant cell lines, both of which were found to have upregulation of the multidrug resistance gene MDR1, suggesting that BV resistance may be mediated in part by increased MDR1 activity rather than CD30 loss [109]. Upregulation of NF-κB activity is responsible for increased MDR1 expression in drug resistant clones [110]. The addition of MDR1 inhibitors to BV treatment may be a potential treatment option for PTCL [109, 111].

#### Pralatrexate

Pralatrexate(PDX), a new antifolate agent that is more potent than methotrexate, is active against T-cell lymphomas. Pralatrexate is a highly selective antifolate drug with high affinity for reduced folate carrier (RFC). RFC is a protein that regulates natural folate uptake and, in tumor cells, is required for purine and pyrimidine biosynthesis [112]. Pralatrexate is the first drug to be approved for patients with relapsed/refractory PTCL based on the PROPEL study. The PROPEL trial study population included patients with all aggressive TCL subtypes, including challenging disease that was partially excluded by other studies, including blastic NTKCL, transformed MF and HTLV-1 ATLL, responses were observed for PTCL in all subtypes. The ORR was 29%(investigator-assessed 39%), with 18% of patients achieving PR and 11% achieving CR or unconfirmed CR (CRu) [113]. After pralatrexate's approval, a phase II trial explored alternating CEOP(cyclophosphamide, etoposide, vincristine, and prednisone) with first-line therapy (each cycle consisted of CEOP (A) with Pralatrexate (B) 30 mg/m2 IV days 15, 22 and 29). However, this study failed to show better results with CHOP than with historical data [114]. During the use of pralatrexate, the overall response rate (ORR) of treated patients was about 30%, which is not ideal for the treatment of T-cell lymphoma. In addition to intrinsic resistance, pralatrexate has cross-resistance with other conventional cytotoxic chemotherapy drugs, and it accumulates over time [113]. Moreover, toxicity of this agent can be significant, the most common grade 3/4 adverse events were thrombocytopenia (32%), mucositis (22%), neutropenia (22%), and anemia (18%) [113, 115], thus we need more research to improve the quality of life of these patients with acceptable comfort indices. The combination appears to overcome the inherent resistance to pratrexin to some extent, and the resistance mechanism of PDX is related to the reduced cellular uptake of PDX and/or the overexpression of DNMT3B. Epigenetic alterations are also thought to play a role in resistance mechanisms. DAC combined with PDX has a synergistic effect, which is expected to improve the clinical efficacy [116]. In addition, pratrexin also has significant synergistic effects with histone deacetylase inhibitors (such as romidispin [117]) and proteasome inhibitors (such as bortezomib [118]).

#### PD-1/PD-L1

Programmed death receptor 1 (PD-1) recognizes ligands such as PD-L1 on tumor cells to evade host immune responses. Inhibition of the programmed cell death ligand 1 (PD-L1) pathway has emerged as a promising strategy for the treatment of tumors [119]. PD-L1 expression ranged from 39 to 100% in NKTCL [30, 120, 121]. Nivolumab is a humanized immunoglobulin G4 monoclonal antibody that targets the programmed death (PD)-1 receptor on T cells and has shown marked antitumor activity, improving survival in a number of solid tumors and hematologic malignancies, including Hodgkin's lymphoma [122, 123]. In a phase Ib study that included R/R hematologic malignancies, the ORR was 40% in patients with PTCL, but no CR was observed [119]. Although Pembrolizumab has been used to treat various subtypes of NHL, there are limited real-world data on the efficacy of Pembrolizumab in patients with NKTCL. Several studies published some clinical trial data, but the sample sizes were small, and some studies showed that PD-1 inhibition with pembrolizumab was a favorable strategy for the treatment of refractory or relapsed NKTCL [124–126]. In addition, PD-L1 mutations and a diverse baseline T-cell receptor (TCR) repertoire have been shown to be potential biomarkers for better selection of NKTCL patients for anti-PD-1 therapy [14]. NKTCL patients can undergo PD-L1 mutation and TCR sequence analysis before receiving immune checkpoint inhibitor therapy to avoid excessive financial burden and reduce adverse events [127].

#### Histone deacetylase inhibitors

Histone deacetylation inhibitors are a class of drugs that can acetylate histone proteins, thereby regulating gene transcription, leading to cell cycle arrest, differentiation and apoptosis [128]. Histone deacetylase inhibitors (HDACis) have long been shown to have anti-tumor activity, and the mechanism is related to acetylation of histones and other proteins involved in tumor suppression, apoptosis, and cell cycle regulation [129]. Romidepsin, a cyclic peptide originally isolated from Chromobacterium violaceum, is a pan-HDAC inhibitor with potent inhibitory activity against selected class 1 HDAC isoforms, such as HDAC-1, -2, and -3, and is a selective and potent bicyclic histone deacetylase inhibitor. First approved by the FDA in 2009 for the treatment of patients with relapsed cutaneous T-cell lymphoma [130], Romidepsin was subsequently approved for the treatment of patients with relapsed/refractory PTCL based on two independent phase 2 clinical trials [131, 132]. The Ro-CHOP Phase III trial was designed to compare the efficacy and safety of standard CHOP versus Romidepsin-CHOP regimens for patients with first-line PTCL.As of December 13, 2019, with a median follow-up of 27.5 months, the Ro-CHOP combination regimen did not improve PFS, remission rates, or OS, nor did it increase the incidence of grade ≥ 3 treatment-related TEAEs in patients with PTCL compared to CHOP treatment. The 6-month, 1-year, and 2-year PFS rates were 67.4%, 67.4%, 67.3%, and 43.2%, respectively, in the Ro-CHOP group, 49.8% and 43.2% in the Ro-CHOP group and 65.9%, 44.3% and 36.3% in the CHOP group, respectively; the 1-year and 2-year OS rates were 78.2% and 63.6% in the Ro-CHOP group and 77.5% and 63.4% in the CHOP group, respectively. However, the high incidence of TEAE in Ro-CHOP treatment limited the intensity of the regimen measured in CHOP. Overall, Ro-CHOP did not demonstrate a satisfactory benefit in patients with previously untreated PTCL [133]. Based on this study, on May 6, 2022, the FDA formally announced that it was withdrawing approval of romidepsin for the indication of PTCL, but that it was still approved for the treatment of cutaneous T-cell lymphoma in patients who have received at least one systemic therapy.

Belinostat, a pan-class 1 and class 2 hydroxamic acid-based HDAC inhibitor, is currently approved for patients with relapsed or refractory PTCL who have received at least one line of previous therapy [134].

Chidamide is another HDACi that has shown monotherapy activity in R/R PTCL [135]. Despite the promising anti-lymphoma activity of histone deacetylase (HDAC) inhibitors, drug resistance is an important clinical problem. Belinostat-resistant cells showed significant cross-resistance to other HDAC inhibitors, including romidepsin, panobinostat, and vorinostat. Consistent with the insensitivity to HDAC inhibitors, resistant cells failed to induce an increase in acetylated histones. Resistance of tumor cells to HDAC inhibitors may involve both "intrinsic" and "acquired" mechanisms. Aberrant expression and modification of signaling molecules lead to the inherent resistance of cancer cells to HDAC inhibitors [128, 136, 137].

#### Inhibitors of the PI3K/Akt/mTOR pathway

In T lymphocytes, activation of the phosphatidylinositol 3-kinase (PI3K)/Akt/ mammalian target of rapamycin (mTOR) pathway in response to exogenous stimuli is known to drive cell survival and clonal proliferation, and PI3K activation is tightly controlled by signals transduced through the TCR complex [138, 139]. Thus, in the presence of stable TCR signaling, costimulatory signaling driven primarily by the PI3K/Akt/mTOR pathway leads to complete T-cell proliferation activation, making inhibition of this pathway and/or TCR signaling a reasonable approach for PTCL therapy [140, 141]. Duvelisib (IPI-145) is an oral inhibitor of the PI3K isoforms PI3Kδ and PI3Kγ, which are thought to be required for full TCR signaling. In an open-label phase 1 study, 16 patients with R/R PTCL and 19 patients with CTCL were treated with duvelisib, with an ORR of 50%, a CR rate of 19%, and a median PFS of 8.3 months [140]. Copanlisib (bay80-6946), a PI3Kα and δ inhibitor, is active in B-cell lymphomas and is FDA approved for the treatment of follicular (B-cell) lymphomas. The activity of copanlisib in T-cell lymphomas is currently being further investigated [142]. Aurora A kinase (AAK) has also recently been shown to play a key function in cell entry into mitosis, and its overexpression has been linked to the development of a number of tumors [143]. This prompted us to explore AAK inhibition as a potential therapeutic strategy for a variety of cancers, including PTCL. Alisertib is a selective AAK inhibitor that has shown promising activity in preclinical models of T—and B-cell lymphomas and in vivo lymphoma models [144, 145]. Despite promising early activity, enrollment was stopped early because of poor odds of superior PFS compared with other agents. The findings, although less encouraging, cannot completely rule out a future role for Alisertib combination therapy.

#### Other drugs

In addition to the above FDA- approved drugs, R/R PTCL can also be treated from other angles. Bendamustine, CCR4 inhibitors [146], ALK inhibitors [147], DNA methyltransferase (DNMT) inhibitors [8], CD138 monoclonal antibody, CD52 monoclonal antibody [148], antiviral drugs, immunomodulators, EZH2 and EZH1 dual inhibitors [149], are drugs that have achieved very encouraging clinical trials in relapsed and refractory PTCL. Furthermore, there is significant emphasis on conducting clinical trials targeting relapsed or refractory T-Cell Lymphomas. In this context, we present a list of ongoing r/r PTCL clinical trials, which encompass novel combinations of established drugs along with newly developed drugs that are not yet available in the market (refer to Table 1).

**Table 1:** Ongoing studies in relapsed/refractory peripheral T-cell lymphoma

NCT number	Study Title	Interventions	phase	Country	*drug target*
NCT05321147	**Safety and Efficacy of Lacutamab in Patients With Relapsed/Refractory Peripheral T-cell Lymphoma That Express KIR3DL2**	Biological: lacutamab	Phase 1	multinational	anti-KIR3DL2 monoclonal antibody
NCT03952078	**A Dose Escalation Study Evaluating CPI-818 in Relapsed/Refractory T-Cell Lymphoma**	Drug: CPI-818	Phase 1	multinational	irreversible Interleukin-2-Inducible T-Cell Kinase (ITK) inhibitor
NCT04447027	**Romidepsin, CC-486 (5-azacitidine), Dexamethasone, and Lenalidomide (RAdR) for Relapsed/Refractory T-cell Malignancies**	Drug: Romidepsin Drug: Lenalidomide Drug: CC-486 (5-azacitidine) Drug: Dexamethasone	Phase 1	United State	DNA methyltransferase (DNMT) inhibitors
NCT04118868	**Pembrolizumab Administered Via the Sofusa® DoseConnect™ in Patients With Relapsed/Refractory Cutaneous T-cell Lymphoma**	Combination Product: Pembrolizumab administered using the Sofusa® DoseConnect™	Phase 1	United State	anti-PD1 antibody
NCT02576496	**Study of Tinostamustine, First-in-Class Alkylating HDACi Fusion Molecule, in Relapsed/Refractory Hematologic Malignancies**	Drug: Tinostamustine	Phase 1	multinational	alkylating deacetylase inhibitor
NCT05079282	**Study of ONO-4685 in Patients With Relapsed or Refractory T Cell Lymphoma**	Drug: ONO-4685	Phase 1	United States	anti-PD-1/CD3 bispecific antibody
NCT05290155	**Anti-CD7 CAR-T Cell Therapy for Relapse and Refractory CD7 Positive T Cell Malignancies**	Drug: anti-CD7 CAR-T cells	Phase 1	China	anti-CD7 CAR-T cells
NCT04774068	**Romidepsin and Parsaclisib for the Treatment of Relapsed or Refractory T-Cell Lymphomas**	Drug: Parsaclisib Drug: Romidepsin	Phase 1	United States	PI3Kdelta inhibitor
NCT05403450	**A Study of Tolinapant in Combination With Oral Decitabine/Cedazuridine and Oral Decitabine/Cedazuridine Alone in Participants With Relapsed/Refractory Peripheral T-cell Lymphoma (R/R PTCL)**	Drug: Tolinapant Drug: Decitabine + Cedazuridine	Phase 1 Phase 2	China	inhibitors of apoptosis proteins
NCT05441761	**Mitoxantrone Hydrochloride Liposomes in Combination With GDP in Relapsed/Refractory PTCL**	Drug: liposomal mitoxantrone hydrochloride, gemcitabine, dexamethasone, and cisplatin	Phase 1 Phase 2	China	liposomal mitoxantrone hydrochloride
NCT05269940	**A Study to Evaluate Activity, Safety and Tolerability of ZX-101A in Relapsed/Refractory Hematological Malignancie**	Drug: ZX-101A	Phase 1 Phase 2	China	PI3Kδ/γ dual target inhibitor
NCT05463263	**A Phase 1/2 Study of STP938 for Adult Subjects With Relapsed/Refractory B-Cell and T-Cell Lymphomas**	Drug: STP938	Phase 1 Phase 2	multinational	cytidine triphosphate synthase 1 (CTPS1) inhibitor
NCT04653649	**CAR T-cells Against CD30 (HSP-CAR30) for Relapsed/ Refractory Hodgkin and T-cell Lymphoma**	Biological: HSP-CAR30	Phase 1 Phase 2	Spain	anti-CD30 CAR-T cells
NCT05559008	**A Umbrella Study in R/R PTCL Guided by Molecular Subtypes**	Drug: Azacitidine Injection Drug: Dasatinib Drug: Linperlisib Drug: Tucidinostat Drug: SHR2554 Drug: Camrelizumab Drug: Apatin	Phase 1 Phase 2	China	therapies guided by molecular subtypes
NCT03011814	**Durvalumab With or Without Lenalidomide in Treating Patients With Relapsed or Refractory Cutaneous or Peripheral T Cell Lymphoma**	Biological: Durvalumab Drug: Lenalidomide Other: Laboratory Biomarker Analysis	Phase 1 Phase 2	United State	anti-PD1 antibody
NCT05138458	**A Study of MT-101 in Subjects With CD5 + Relapsed/Refractory TCL (IMAGINE)**	Biological: MT-101 Other: MT-101 + Conditioning (Lymphodepleting) Chemotherapy	Phase 1 Phase 2	United States	anti-CD5 CAR-T cells
NCT05627856	**A Study of GNC-038 Injection in Patients With Relapsed or Refractory NK/ T-cell Lymphoma, AITL, and Other NHL**	Drug: GNC-038	Phase 1 Phase 2	China	tetraspecific antibody that binds four tumor-associated targets—CD3, 4-1BB, PD-L1 and CD19
NCT05182957	Clinical Study on Anti-PD-1 Plus Lenalidomide and Azacitidine in Relapsed/Refractory Peripheral T-cell Lymphoma	Drug: Anti-PD-1 monoclonal antibody Drug: Lenalidomide Drug: Azacitidine	Phase 2	China	anti-PD-1 monoclonal antibody
NCT05140382	AZD4573 as Monotherapy or in Combinations With Anti-cancer Agents in Patients With r/r PTCL or r/r cHL	Drug: AZD4573	Phase 2	multinational	CDK9 inhibitor
NCT04512534	Sintilimab Combined With Chidamide in Treating Peripheral T Cell Lymphoma	Drug: PD-1 antibody + HDAC inhibitor	Phase 2	China	PD-1 antibody + HDAC inhibitor
NCT04329130	**Chidamide Combination With Lenalidomide in Patients With Relapsed or Refractory Peripheral T-cell Lymphoma**	Drug: Chidamide, Lenalidomide	Phase 2	China	HDAC inhibitor
NCT04083495	**CD30 CAR for Relapsed/Refractory CD30 + T Cell Lymphoma**	Biological: ATLCAR.CD30 T cells Drug: Fludarabine Drug: Bendamustine Drug: Cyclophosphamide	Phase 2	United State	anti-CD30 CAR-T cells
NCT05495100	**A Single-arm, Multicenter, Prospective Clinical Study of Mitoxantrone Liposome Combined With Chidamide and Azacitidine in the Treatment of Relapsed and Refractory Peripheral T-cell Lymphoma**	Drug: Mitoxantrone liposome、Chidamide、Azacitidine	Phase 2	China	Mitoxantrone liposome
NCT04105010	**Assessing An Oral Janus Kinase Inhibitor, AZD4205 as Monotherapy in Patients Who Have PTCL (JACKPOT8)**	Drug: AZD4205	Phase 2	China	Oral Janus Kinase inhibitor
NCT05313243	**Pembrolizumab and Brentuximab Vedotin in Subjects With Relapsed/Refractory T-cell Lymphoma**	Drug: Brentuximab vedotin Drug: Pembrolizumab	Phase 2	United State	anti-CD30 monoclonal antibody, anti-PD-1 monoclonal antibody
NCT04984837	**Study of Lacutamab in Peripheral T-cell Lymphoma**	Drug: Lacutamab Drug: Gemcitabine Drug: Oxaliplatine	Phase 2	multinational	anti-KIR3DL2 monoclonal antibody
NCT02588651	**A Phase II Study of Single Agent Brentuximab Vedotin in Relapsed/Refractory CD30 Low (< 10%) Mature T Cell Lymphoma (TCL)**	Drug: Brentuximab vedotin	Phase 2	United State	anti-CD30 monoclonal antibody
NCT04217317	**CPI-613 in Combination With Bendamustine in Patients With Relapsed/Refractory T-Cell Non-Hodgkin Lymphoma**	Drug: CPI 613 Drug: Bendamustine	Phase 2	United States	a lipoate analogue that inhibits pyruvate dehydrogenase (PDH) and α-ketogluterate dehydrogenase (KGDH)
NCT05059912	**CD7 CAR T-cell for R/R CD7 + T Cell Lymphoma**	Biological: Humanized CD7 CAR-T cells	Phase 2	China	anti-CD7 CAR-T cells
NCT04763616	**Study of Isatuximab and Cemiplimab in Relapsed or Refractory Natural Killer/T-cell Lymphoid Malignancy (ICING)**	Drug: Isatuximab Drug: Cemiplimab	Phase 2	Korea	anti-CD38 monoclonal antibody, PD-1 antibod
NCT04296786	**Sintilimab Plus Chidamide in the Treatment of Relapsed and Refractory Cutaneous T-cell Lymphoma: a Multicenter Phase II Study**	Drug: Sintilimab Drug: Chidamide	Phase 2	China	PD-1 antibod
NCT04414163	**A Study of IMC-001 in Subjects With Relapsed or Refractory Extranodal NK/T Cell Lymphoma, Nasal Type**	Drug: IMC-001	Phase 2	Korea,	PD-1 antibod

## Conclusion

To date, most PTCL subtypes are aggressive and chemotherapy-resistant, and their prognosis remains poor. Multiple mechanisms, such as tumor heterogeneity, tumor microenvironment, and signaling pathways, contribute to PTCL resistance. Over the past few years, considerable efforts have been made to identify novel molecular targets and deregulated molecules in the oncogenic pathway. Many ongoing clinical trials are exploring other targeted drugs, novel cell therapies, and immunotherapies. As precision medicine catches up with this disease, we are likely to see new treatments that can overcome tumor resistance and thus improve the treatment efficacy of PTCL.

## Data Availability

The data and materials supporting the study are available upon request by writing to the corresponding authors.

## Abbreviations

PTCL: Peripheral T-cell lymphoma
r/r PTCL: Relapsed or refractory peripheral T-Cell Lymphomas
NHL: Non-Hodgkin's lymphomas
WHO: World Health Organization
WHO-HAEM5: WHO classification of haematolymphoid tumors
TME: Tumor microenvironment
CTCL: Cutaneous T-cell lymphoma
ALCL: Anaplastic large cell lymphoma
AITL: Angioimmunoblastic T-cell lymphoma
T-ALL/LBL: T lymphoblastic leukemia/lymphoma
ETP-ALL: Early T-cell precursor lymphoblastic leukemia
sEBV + TNHL: Systemic EBV + T-cell lymphoma
ENKTL: Extra-nodal NK/T-cell lymphoma
ATLL: Adult T-cell leukemia/lymphoma
CCR4: C-C chemokine receptor 4
ALCL: Anaplastic large cell lymphoma
PD-L1: Programmed cell death ligand I
OS: Overall survival
Tregs: Regulatory T cells
MDR: Multidrug resistance
ABC: ATP-binding cassette
P-gp: P-glycoprotein
MRP-1: Multidrug resistance-related protein 1
BCRP: Breast cancer resistance protein
LRP: Lung resistance protein
EPI: Epirubicin
NF-κB: Nuclear factor κB
MAPK: Mitogen-activated protein kinase
SMILE: Steroids, methotrexate, ifosfamide, L-asparaginase, and etoposide
TFH: Follicular helper T cell
MF: Mycosis fungoides
ENKTCL: Extranodal NK/ t cell lymphoma
T-LBL/ALL: T lymphoblastic lymphoma/leukemia
BV: Brentuximab Vedotin
MMAE: Monomethyl lauristatin E
NLG: Nordic Lymphoma Group
PDX: Pralatrexate
RFC: Reduced folate carriers
CRu: Unconfirmed CR
CEOP: Cyclophosphamide,etoposide, vincristine, and prednisone
ORR: Overall response rate
PD-1: Programmed death receptor 1
TCR: T-cell receptor
HDAC: Histone Deacetylase Inhibitors
PI3K: Phosphatidylinositol 3-kinase
AAK: Aurora A kinase
DNMT: DNA methyltransferase
